# Simulation and experimental study of a cold atmospheric pressure plasma and comparison of efficiency in boosting recombinant Endoglucanase II production in *Pichia pastoris*

**DOI:** 10.1371/journal.pone.0303795

**Published:** 2024-05-21

**Authors:** Zeinab Kabarkouhi, Saeed Hasanpour Tadi, Hadi Mahmoodi, Seyed Omid Ranaei Siadat, Sareh Arjmand, Babak Shokri

**Affiliations:** 1 Laser and Plasma Research Institute, Shahid Beheshti University, Tehran, Iran; 2 Protein Research Center, Shahid Beheshti University, Tehran, Iran; Satyawati College, University of Delhi, INDIA

## Abstract

Recombinant proteins are essential in various industries, and scientists employ genetic engineering and synthetic biology to enhance the host cell’s protein production capacity. Stress response pathways have been found effective in augmenting protein secretion. Cold atmospheric pressure plasma (CAP) can induce oxidative stress and enhance protein production. Previous studies have confirmed the applicability of CAP jets on Phytase and green fluorescent protein (GFP) production in *Pichia pastoris* hosts. This study investigates the effect of CAP treatment on another valuable recombinant protein, Endoglucanase II (EgII), integrated into the *Pichia pastoris* genome. The results demonstrated that plasma induction via two different ignition modes: sinusoidal alternating current (AC) and pulsed direct current (DC) for 120, 180, and 240 s has boosted protein secretion without affecting cell growth and viability. The AC-driven jet exhibited a higher percentage increase in secretion, up to 45%. Simulation of plasma function using COMSOL software provided a pattern of electron temperature (T_e_) and density distribution, which determine the plasma cocktail’s chemistry and reactive species production. Furthermore, electron density (n_e_) and temperature were estimated from the recorded optical spectrum. The difference in electron properties may explain the moderately different impressions on expression capability. However, cell engineering to improve secretion often remains a trial-and-error approach, and improvements are, at least partially, specific to the protein produced.

## 1. Introduction

Plasma is generated by applying an electric field, which ionizes the gas and leads to the formation of a discharge. This process results in the formation of a cluster of free electrons and positive ions that react with feeding gas and surrounding air molecules [1]. The complex reactions happening in plasma create a chemically active medium, reacting with the surrounding environment and desired target. The high reactivity of plasma is the primary reason for its wide range of applications across various fields [2, 3], including biomedical applications. Cold atmospheric pressure plasma (CAP) has made significant strides in the field of biology since the mid-1990s. Low-temperature CAP has demonstrated remarkable results in various areas, such as blood coagulation, cancer treatment, skin rejuvenation, and wound healing [4–8]. One popular configuration of atmospheric plasma production is CAP jets (CAPJ) based on dielectric barrier discharges (DBD) [9–11]. While most DBD jets are ignited by alternating current (AC) power supplies, researchers have also established the safe operation of pulsed direct current (DC) jets [12].

Gaining a comprehensive understanding of gas discharge and fluid flow dynamics is crucial for the effective utilization of plasma jets across applications. 2D finite element method (FEM) simulations can provide valuable insight into the gas discharge and fluid flow dynamics within the plasma jet [13, 14]. Furthermore, experimental analysis of CAPJ can leverage optical emission spectroscopy (OES), a straightforward and cost-effective plasma diagnostic technique offering reasonably precise outputs.

In this study, we investigate the effects of two operational modes of a plasma jet on the production of recombinant Endoglucanase II (EgII) in a recombinant yeast strain, with the aim of exploring the potential of CAP as an assistant in the recombinant protein industry. Previous studies have shown the efficiency of CAP in augmenting the production of green fluorescent protein (GFP) and Phytase in recombinant yeast, and the effects of plasma on the expression of some genes were investigated [15, 16].

According to market research, the global industrial enzymes market size was valued at USD 6.63 billion in 2022 and is projected to reach USD 11.02 billion by 2030 [17]. The global enzymes market is expected to grow significantly, with North America leading the market. The hydrolase segment, which includes cellulases, amylases, pectinases, lipases, phytases, and proteases, dominates the enzyme market due to its various applications in several industry verticals [18]. Cellulose, the most abundant natural polymer, is broken down by cellulases through the hydrolysis process in various industries like foods and beverages, detergents, agriculture, paper, and textiles [19, 20]. Furthermore, the demand for biofuels as a replacement for fossil fuels is increasing, and this requires a significant supply of cellulases for the new era of energy resources. Cellulases are naturally produced in various microorganisms, such as *Trichoderma reesei* fungi and *Bacillus subtilis* bacteria [21, 22]. Any potential method that may decrease the cost of the cellulase production process in the aforementioned industries is highly appreciated. *Trichoderma reesei* fungi produce EgII, a key enzyme with high catalytic efficiency and activity at pH 4–6.7 [23, 24]. Recombinant endoglucanase, with improved thermal stability and mass production capabilities, is a significant development for increasing commercial efficiency and providing great economic value in cellulase production [25].

In this study, we aimed to investigate different modes of DBD plasma jet operation for the treatment of recombinant *Pichia pastoris* yeast, expressing EgII enzyme. 2D FEM simulation investigations were performed by COMSOL software to compare the plasma parameters between ignitions by sinusoidal or DC-pulsed power supplies. Moreover, OES spectrum was utilized to estimate electron temperature (T_e_) and density in AC mode of the plasma jet.

The EgII gene from *Trichoderma reesei* has been cloned into the yeast *Pichia pastoris* for heterologous production host previously in our research group [24]. In the present work, we treated the recombinant *Pichia pastoris* yeast carrying the EgII gene with a single plasma jet turned on by two different power supplies. The secreted proteins in collected supernatants were quantified using SDS-PAGE and Bradford tests, and the enzyme activity was assayed through a reducing sugars colorimetric analysis.

The results of this study could provide valuable insights into the use of CAP in the recombinant protein industry.

## 2. Methods

### 2.1. The plasma jet description and specification

**Fig 1:**
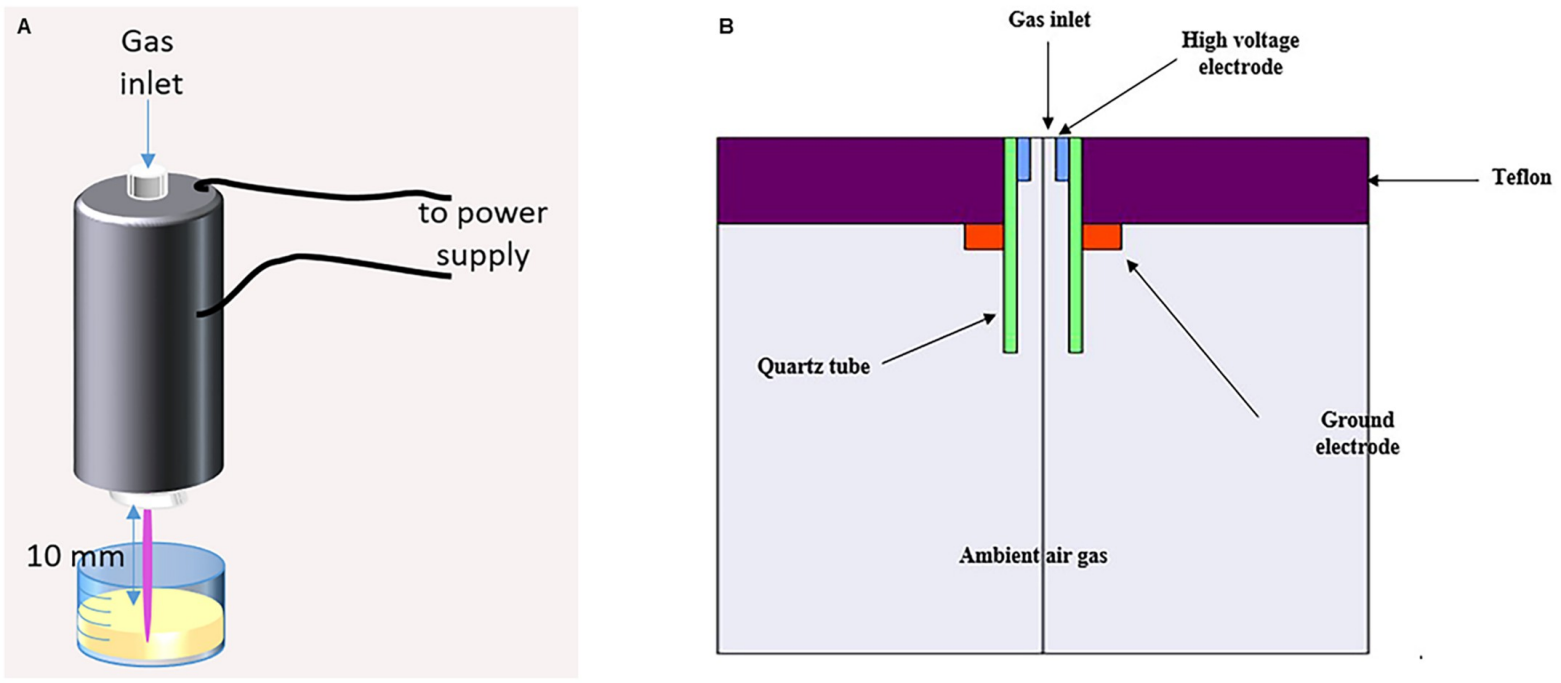
The plasma jet 2D schematic (A) experimental setup, and (B) simulation modeling.

A CAP jet, based on DBD, was applied (Fig 1A). The jet coaxial configuration is composed of two electrodes, a ring, and a rod electrode, covered by a quartz tube as a dielectric barrier. 99.999% pure helium is injected into the cavity interior of the insulated jet with three standard liters per minute flow rate and is ionized to break down by an electric field induction. Here, two different power supplies were examined as electric voltage applications: an AC power supply with 4 kV peak-to-peak voltage and 20 kHz frequency; and a DC pulsed power supply with 10 kHz frequency, DC voltage amplitude up to 10.0 kV at a duty cycle of 2%. The generated plasma is propelled outwards and into the ambient air through the jet nozzle and forms the plasma plume. The plasma plume, sometimes called effluent, is about 2 cm long and 10 mm in diameter and is ready for action on target [26].

#### 2.1.1. Simulations by COMSOL software

The gas discharge and fluid dynamics were simulated using COMSOL software based on the governing equations reported by Nguyen, Tam, et al. (Fig 1B). The laminar fluid flow in the plasma jet was modeled by solving the incompressible Navier-Stokes equations, under the assumption that the gas compressibility term is negligible at the low velocities considered [14, 27]. This incompressible flow assumption is valid since the uniform flow streamlines indicate the absence of vortex currents or turbulence at the jet outlet. The electric discharge physics was modeled by solving the drift-diffusion equations for charged species transport, the continuity equations for charge conservation, the momentum equations for charged species velocities, and the Poisson’s equation for the electric potential distribution.

As a matter of simplicity, only the helium reactions including He (2^3^S_1_) = He*, He_2_ (α^3^Σ_u_) = He_2_*, He^+^, He_2_^+^ have been taken into account. Table 1 illustrates the electron impact reaction and the chemistry of helium [13].

**Table 1:** Helium chemistry used in the simulation.

Index	Reaction	Coefficient	Activation (eV)
R1	e + He→e + He	BOLSIG+	-
R2	e + He→e + He*	BOLSIG+	19.8
R3	e + He→2e + He^+^	BOLSIG+	24.6
R4	e + He*→2e + He^+^	BOLSIG+	4.78
R5	e + He_2_*→2e + He_2_^+^	1.268 × 10^−18^ T_e_^0.71^ exp(−3.4/T_e_)	3.4
R6	2He*→e + He + He^+^	4.5e-16	-15
R7	2He*→e + He_2_^+^	2.03e-15	-19.6
R8	He^+^+2He→He_2_^+^+He	1e-43	-
R9	He* + 2He→He_2_* + He	1.3e-45	-
R10	e + He*→e + He	2.9e-15	-19.8
R11	2e + He+→e + He*	5.12 × 10^−39^ T_e_^−4.5^	-4.78
R12	e + He+→He	2e-18	-24.6
R13	e + He_2_^+^→He* + He	5.386 × 10^−13^ T_e_^−0.5^	-0.2
R14	e + He_2_^+^→2He	9e-15	-20

According to jet parameters (jet length, gas inlet velocity), electric discharge and chemical reaction time processes in plasma simulations have dimensions of nanoseconds or microseconds, while gas flow occurs within fractions of a second. Consequently, plasma particles experience stable fluid during an electric discharge. Thus, the stationary state solver was applied for fluid flow simulation, and time-dependent solvers were used for electrical discharge and chemistry simulation.

#### 2.1.2. OES and the line-ratio method for electron temperature and density estimation

The line-ratio method, applied to the OES spectrum intensities, is based on the dependency of line intensity on electron density (n_e_) and temperature (T_e_) through the relative intensities of two selected emission lines [28]. The line-ratio method is applied in partial local thermal equilibrium (pLTE) plasmas, assuming the validity of the Saha-Boltzmann equation for a limited range of excited-level populations. One can say that according to a survey by Şahin et al. and a modified collisional-radiative (CR) model by Akatsuka, He emission lines starting from n = 3 levels may satisfy the criteria for using the line-ratio method for the determination of approximate T_e_ [29, 30]. Briefly, the excitation kinetics CR model is adopted for atmospheric pressure plasmas where collisional or quenching processes are dominant. Furthermore, it’s assumed that photo-absorption is negligible, meaning the plasma is optically thin. In the current study, the line ratio method was performed on the spectrum of AC-driven plasma plumes. The line pair was selected to be 728.1 nm/706.5 nm of He I emission lines according to Şahin et al.’s preferred alternative of singlet states/triplet states, which has been proven to be T_e_ sensitive [31].

The equation applied for the T_e_ calculation was as follows, by the assumption of a Boltzmann distribution for the population of excited states:

$$k_{B}T_{e}=(E_{m}-E_{i}){\left(ln\frac{A_{mn}g_{n}I_{ij}\lambda_{ij}}{A_{ij}g_{i}I_{mn}\lambda_{mn}}\right)}^{-1},$$ (1)

where *E*_*m*_, *E*_*i*_ are the energies, *k*_*B*_ is Boltzmann constant (8.617×10^−5^ eV/K), *g*_*n*_, *g*_*i*_ are statistical weights of the upper levels, and *I*_*ij*_, *I*_*mn*_ are the measured intensities at the wavelength *λ*_*ij*_ (706.5 nm) and *λ*_*mn*_ (728.1 nm) respectively.

To have an estimation of n_e_, the ratio of a line pair must be taken between a neutral atom or molecule and the ionized form. The recorded spectrum of our plasma doesn’t include the noticeable ionized form of helium; therefore, nitrogen molecule peaks are considered for pair selection. The selected nitrogen lines were 357.6 nm and 391.1 nm from the second positive system of N_2_ and the first negative system band of N_2_^+^, respectively. Applying the Saha-Boltzmann equation to the line intensities ratio with the assumption of the Boltzmann distribution of excited states, n_e_ can be calculated employing estimated T_e_, as below:

ne=2(2πmekBTe)32h3I357.6A391.1g391.1λ357.6I391.1A357.6g357.6λ391.1e−Eion−E391.1+E357.6kBTe (2)

where *m*_*e*_ is electron mass, *E*_*ion*_ the ionization of nitrogen molecule (15.58 eV) and h Planck constant.

### 2.2. Experimental setup

#### 2.2.1. Plasma treatment procedure

The recombinant *Pichia pastoris* yeast strain expressing recombinant EgII was obtained from our previous study [24, 32]. Briefly, a gene encoding endo-1,4-beta-glucanase (GenBank: JF340120.1) was adapted to the codon bias of *Pichia pastoris* genes and cloned in the pPink-αHC plasmid, directly downstream of an α-factor secretion signal, and under the control of a methanol-inducible promoter, AOX1. The recombinant plasmid was propagated in DH5α strain of *Escherichia coli* cells and transformed in the PichiaPink^™^ expression system strain 2 (ade2, pep4). The yeast cells were initially cultured in YPG medium (1% (w/v) yeast extract, 2% (w/v) peptone, 2% (v/v) glycerol). Once the yeast cells reached a suitable growth phase, they were transferred to YPM medium (1% (w/v) yeast extract, 2% (w/v) peptone, 0.5% methanol (v/v)) for the induction of recombinant protein expression under the control of AOX1 promoter. The recombinant protein expression was sustained for a consecutive period of three days through a regimen of daily 1% methanol feeding. The growth conditions were 28–30°C with 250 rpm shaking.

The plasma treatment was performed by the DBD plasma jet. Fig 1A illustrates a schematic of a plasma jet as in experiments.

Recombinant *Pichia pastoris* yeast cells were cultured in YPG medium to reach the desired optical density (OD). Equal concentrations of culture were inoculated to YPM medium. Exactly before the expression induction by methanol, the cell culture of 15 milliliters was treated by CAPJ. A distance gap of 10 mm was kept between the jet nozzle and culture interface as a target in a glass beaker. The beaker is on a magnetic stirrer during the plasma treatment to ensure a homogenous treatment.

#### 2.2.2. Cell health and protein/enzyme quantifications

The cell growth and viability were monitored during the three days of methanol feeding via MTT assay and OD_600_ measurements. The slightly modified colorimetric MTT protocol for yeast according to Ryu et el. is described in our previous paper in detail [33].

The secreted protein content in the supernatant was quantified via Bradford reagent (Navandsalamat, Iran) in comparison with BSA standards [34]. To enhance the accuracy of the Bradford assay for quantifying the recombinant protein of interest in the supernatant, we implemented a normalization strategy using a non-recombinant strain of *Pichia pastoris* that was cultured under similar conditions and with comparable cell concentrations as a control. Furthermore, a visual analysis of the produced protein was performed by sodium dodecyl-sulfate polyacrylamide gel electrophoresis (SDS-PAGE), followed by gel staining with Coomassie blue dye. Densitometry analysis was performed using ImageJ to complement total protein quantification. BSA standards enabled the approximation of sample band intensities. The gel image was inverted to 8-bit grayscale. Individual rectangular regions of interest (ROIs) were defined for each band and intensity histograms were plotted. Peak areas were measured and used to generate a calibration curve by linear regression. This enabled the estimation of relative sample concentrations. While densitometry analysis enables the estimation of relative band intensities, sources of potential error include variations in ROI selection between analyses and differences in staining efficiency between gels. To minimize variability, ROIs were defined with consistent dimensions and positions across samples, and replicate analyses were performed. However, these semi-quantitative estimates should be considered approximations with uncertainties, which provide complementary data to the spectroscopic assay.

The enzymatic activity of EgII was measured by a reducing sugars colorimetric common analysis dating back to 1921 [35]. Carboxymethyl cellulose (CMC) 1% was used as a cellulose derivative with enough accessible β-Glycosidic bonds as enzyme substrate for hydrolysis into glucose monomers at 50°C for 30 minutes. The yellow 3,5-Dinitrosalicylic acid (DNS) serves as a detector of produced glucose units, called reducing sugars. DNS is reduced by the free carbonyl group of glucose in a boiling water bath for 5 minutes and forms 3-amino-5-nitrosalicylic acid with a red-brown color. The absorbance of collected supernatants and glucose dilution standards (in sodium citrate buffer 2 mM) at 540 nm provides the content of produced glucose. The resulting quantities along with enzyme concentrations from the Bradford assay contribute to enzyme activity plots.

## 3. Results

### 3.1. Plasma specification

#### 3.1.1. Modeling results

**Fig 2:**
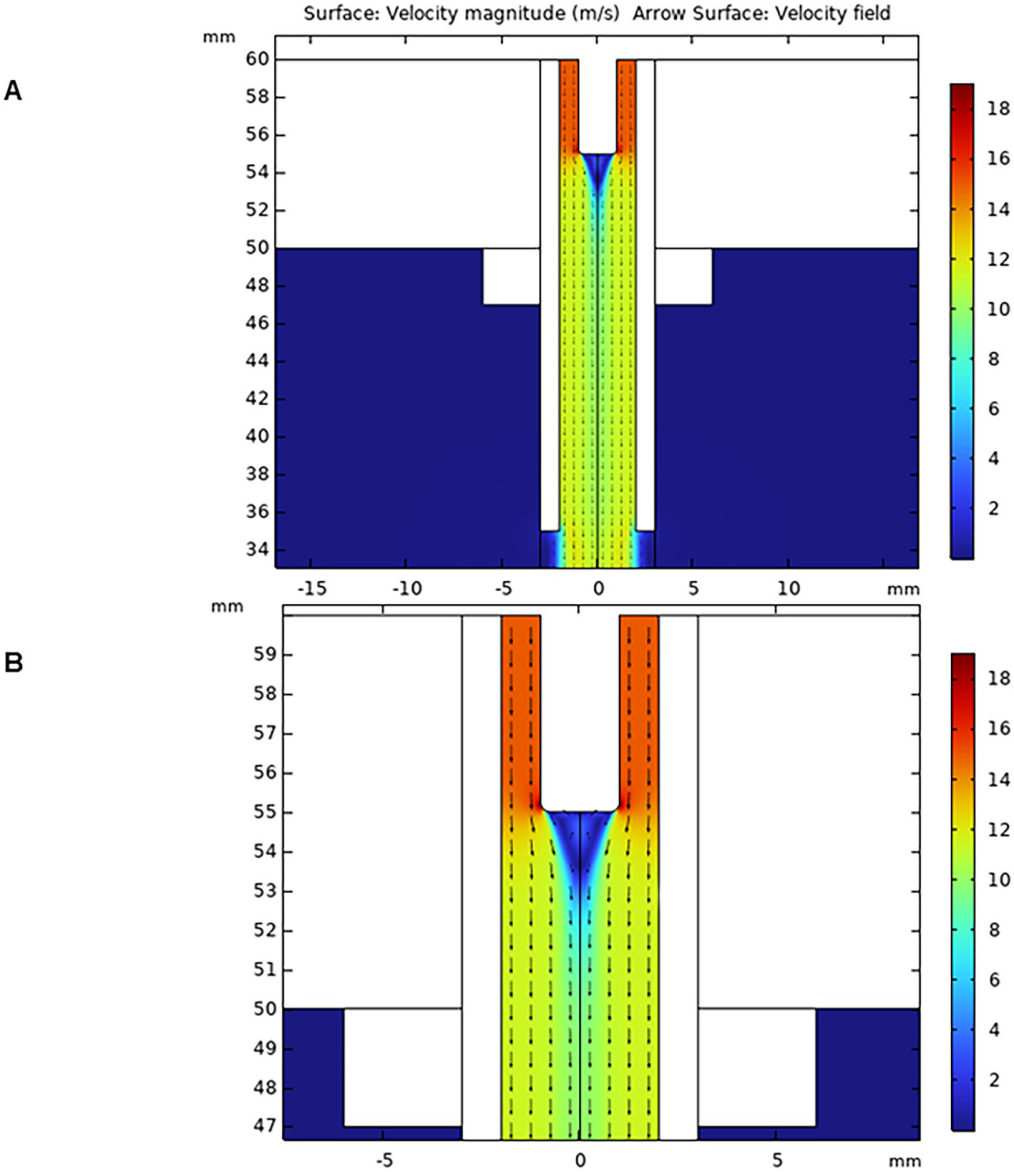
2D surface plot of plasma jet velocity distribution. (A) Normal view (B) magnified view representing flow lines.

The fluid simulation results, regardless of power supply properties, are presented in Fig 2A, with an enlarged view available in Fig 2B. It was observed that the fluid head speed reaches 15 m/s inside the jet and close to the rod electrode, and 10 m/s in the jet outlet. The speed decreases as a result of adiabatic expansion while exiting the jet. The velocity profile is symmetric, and flow lines are parallel to each other, resulting in laminar flow.

**Fig 3:**
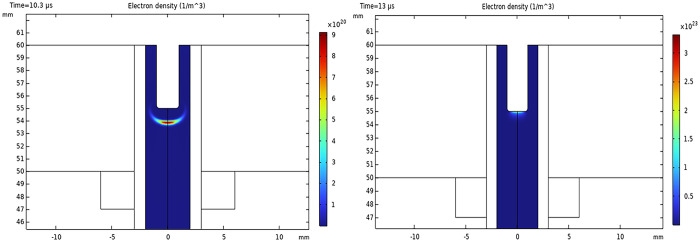
Surface pattern for n_e_ distribution. (A) The plasma jet runs with a sinusoidal power supply, and (B) with a pulsed DC power supply.

Fig 3A and 3B show a two-dimensional pattern of plasma density for sinusoidal AC and pulsed DC modes, respectively. The n_e_ in sinusoidal mode is 10^20^−10^21^ m^-3^ (10^14^−10^15^ cm^-3^), while it reaches 10^22^−10^23^ m^-3^ in pulsed DC mode. Higher electron densities are expected in DC mode due to a higher applied voltage of 10 kV.

**Fig 4:**
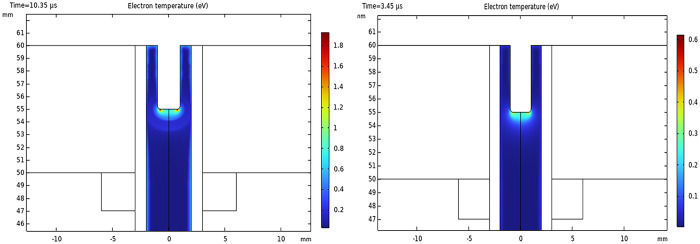
Surface pattern for T_e_ (eV) distribution. (A) The plasma jet runs with a sinusoidal power supply, and (B) with a pulsed DC power supply.

Fig 4 shows the T_e_ distributions for AC and pulsed DC modes. According to the observation, there is a near-equal distribution of T_e_, leaving out the inner electrode vicinity. Average T_e_ is 1.2 eV and 0.6 eV for AC and DC operations, respectively. The highest temperatures are observed on the edges of the rod electrode.

**Fig 5:**
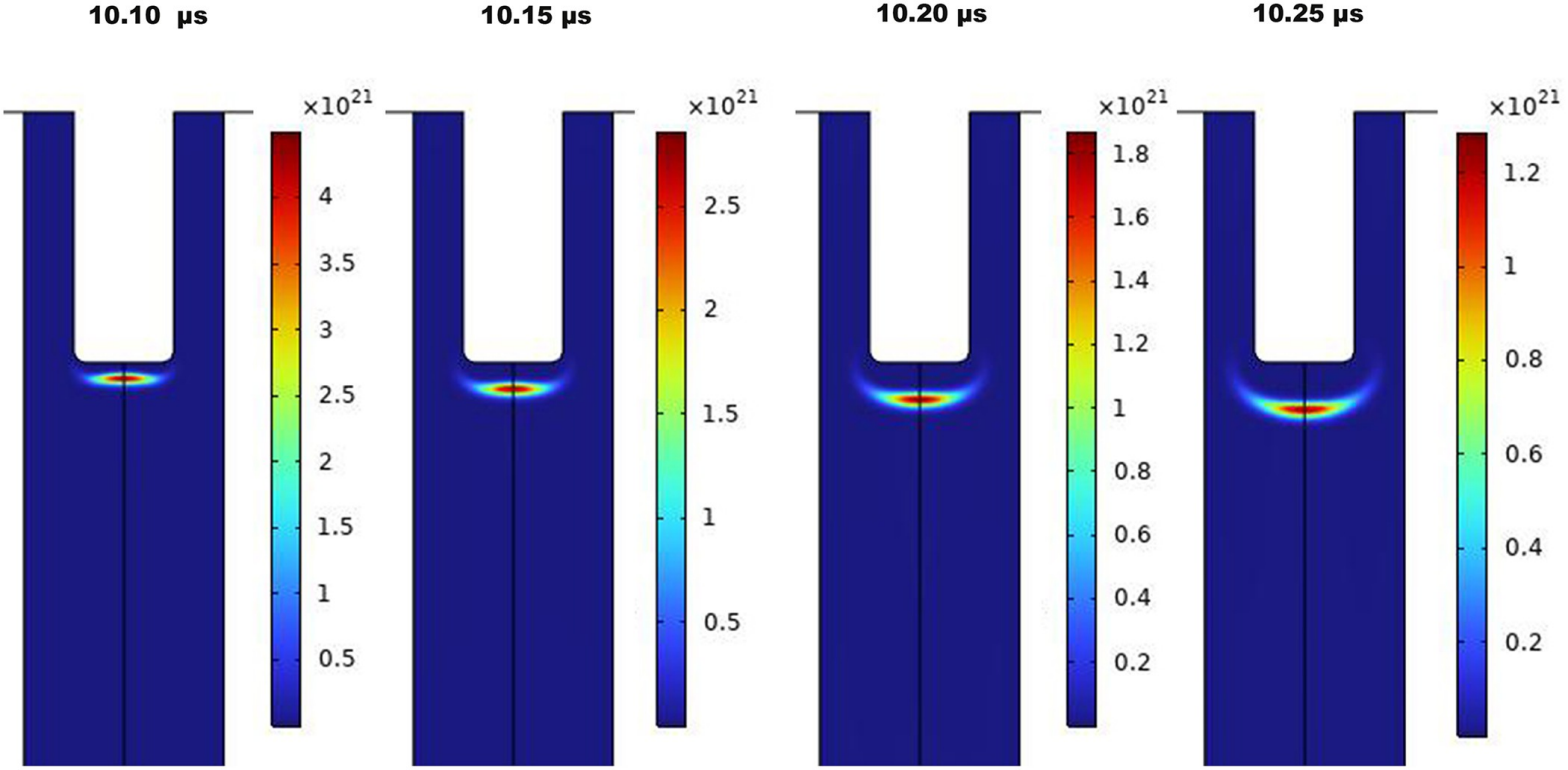
The simulated spatial and temporal distribution of n_e_ of AC-driven jet.

Fig 5 represents the spatial and temporal evolution of the n_e_ of AC discharge, which manifests the movement of the ionization wave front in a cycle. The development of ionization wave fronts, or, plasma bullets, can be clearly observed in the snapshots. Interestingly, the conventional streamer head shape is evidently noticeable in the continuous AC ignition.

**Fig 6:**
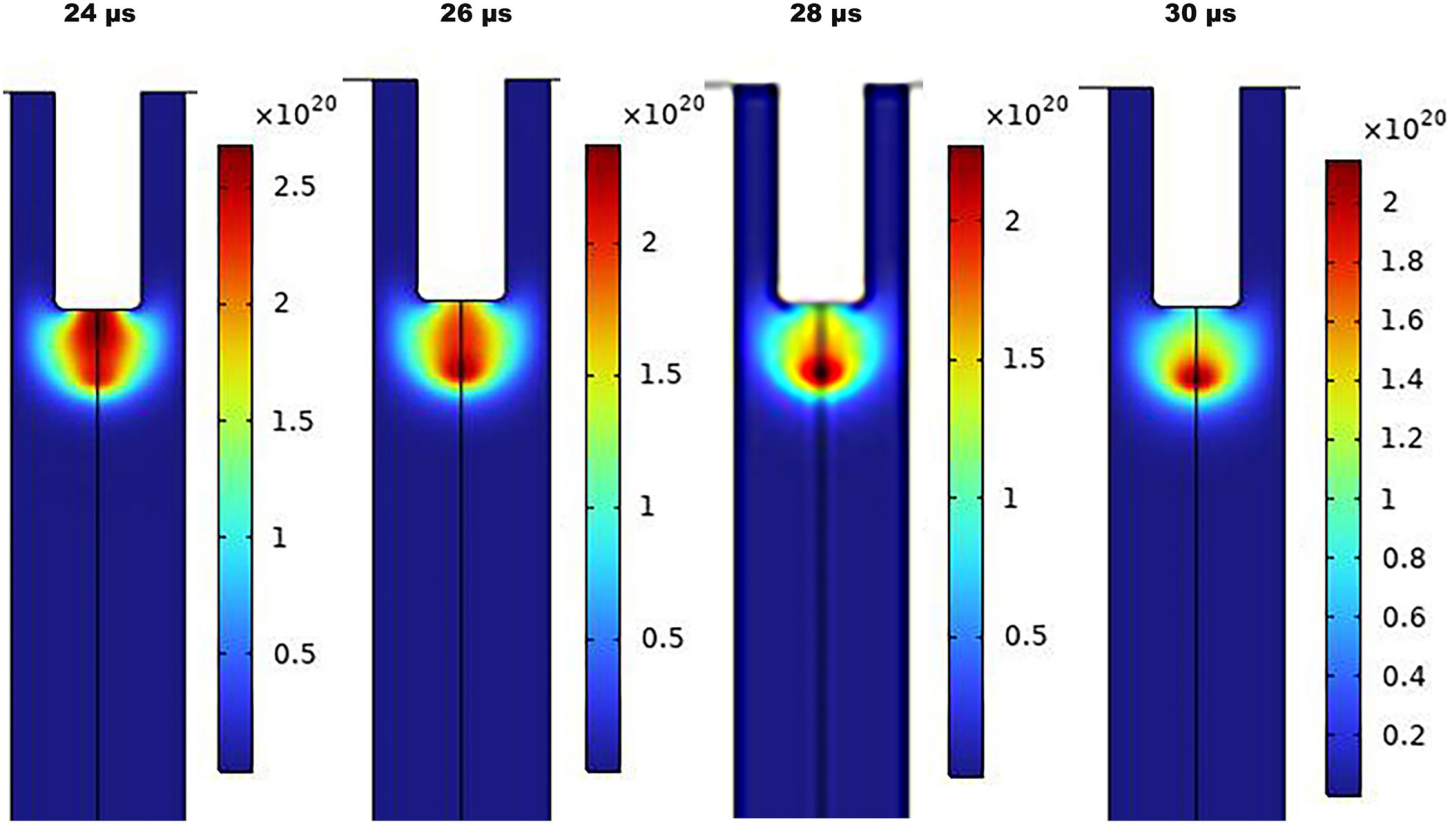
The simulated spatial and temporal distribution of n_e_ of pulsed DC-driven jet.

A similar n_e_ distribution for pulsed DC discharge is demonstrated in Fig 6. The larger area of bullets with electron bunches may explain the averaged higher n_e_ of pulsed operation. The circular shape of the ionization front may result from the self-propagation of electron avalanches in the absence of electric force as the voltage pulse is terminated in studied time scales.

#### 3.1.2. Electron temperature and density determination using OES spectrum

**Fig 7:**
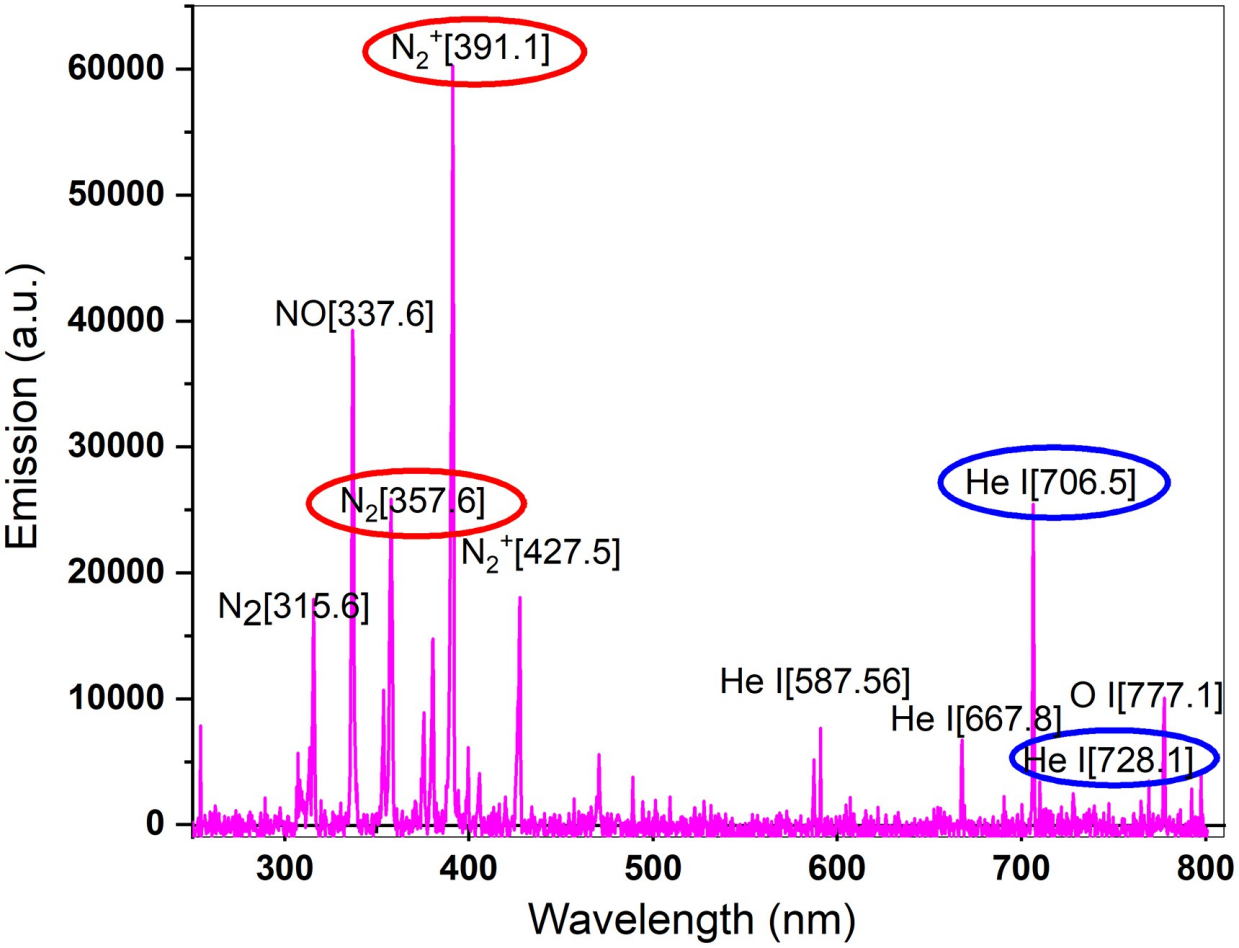
Optical emission spectrum and some designated peaks according to NIST database.

OES is a frequent method for plasma diagnostics, with the advantages of being non-intrusive, uncomplicated, and inexpensive. The liberated energetic electrons during discharge occurrence collide with atoms and molecules and may excite some bound electrons to higher levels. As the excited states are unstable, excited, jumped-up electrons make transitions to lower energy levels to relieve their energy in the form of photons of light. The emitted radiation, corresponding to the distinguished transitions between discrete electronic levels, is recorded by standard optical spectrometers in the ultraviolet (UV), visible, and near-infrared (NIR) ranges, typically 200–1100 nanometers. Multiple data concerning plasma characteristics can be extracted based on the recorded spectrum, including T_e_ [36]. Additionally, it demonstrates information on the present species including reactive oxygen and nitrogen species (RONS) in plasma and their abundance, as we conducted in our previous study [15]. In fact, the emission lines provide clues concerning reactions occurring in the plasma. OES spectrum is observed in Fig 7 with a brief designation of lines. The peaks employed in the line ratio method assuming pLTE for T_e_ and density are assigned by blue and red ovals, respectively.

As mentioned above, the selected ratios were He lines at 706.5 nm and 728.1 nm for T_e_ rough calculation and neutral and ionized molecular nitrogen at 357.6 nm and 391.1 nm for n_e_ estimation using calculated T_e_ for plasma jet formed in DBD configuration at atmospheric pressure. The quantities of statistical weights, Einstein coefficients, and levels’ energy for the He I line pair are extracted from the NIST spectra databases.

The T_e_ estimation, obtained by the line ratio for singlet and triplet states with the data listed in Table 2, resulted in T_e_ being 1.4851 eV. The calculated T_e_ is in good agreement with the T_e_ in simulations of AC mode (Fig 5).

**Table 2:** The selected He I electronic transition characteristics.

Transition	Wavelength (nm)	Statistical weight of upper-level	Energy of upper level (eV)	Einstein coefficient (s^-1^)
2^3^P-3^3^S	706.5	3	22.7185	1.5474 x10^7^
2^1^P-3^1^S	728.1	1	22.9203	1.8299 x10^7^

Eq 2 was utilized for n_e_ determination and led to n_e_ = 10^14^ cm^-3^. The required data corresponding to molecular nitrogen (Table 3) for n_e_ estimation were taken from the PLASUS SpecLine Database and the precious tables of Gilmore et al. [37] Interestingly, the maximal modeled n_e_ is in good agreement with the calculated density here from Eq 2, which may demonstrate close to reality assumptions.

**Table 3:** The selected neutral and ionized molecular nitrogen transitions’ characteristics.

Transition	Wavelength (nm)	Statistical weight of upper-level	Energy of upper level (eV)	Einstein coefficient (s^-1^)
N_2_(*C*^3^ *Π*_*u*_ − *B*^3^ *Π*_*g*_)	357.6	1	11.05	8.84 x10^6^
N_2_^+^($B^{2}\Pi_{u}^{+}-X^{3}\Pi_{g}^{+}$)	391.1	1	3.16	1.14 x10^7^

### 3.2. CAP impressions on cell growth and viability, protein production, and enzyme activity

**Fig 8:**
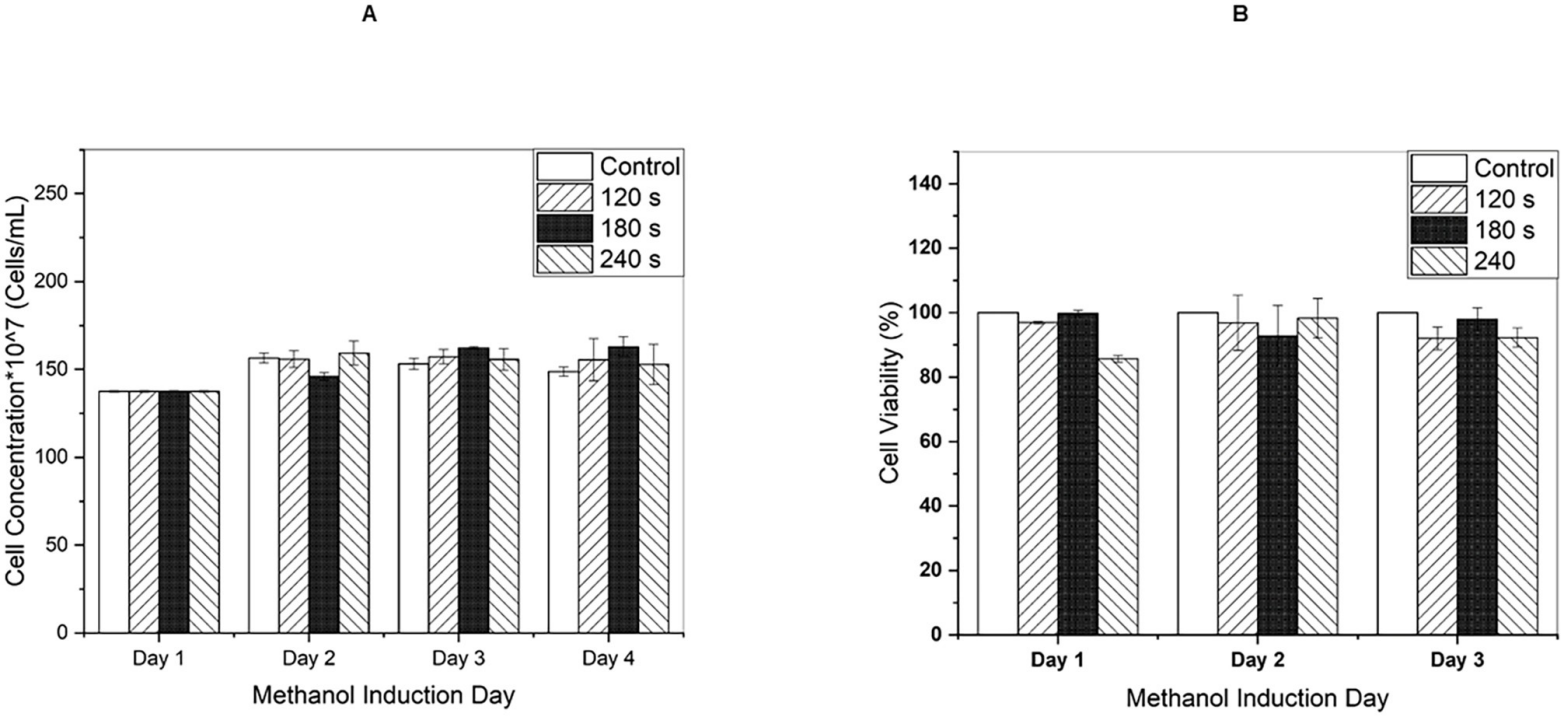
CAP impression after three exposure durations on (A) Cell growth by OD_600_ measurements and (B) viability via MTT assay. The error bars represent standard deviations.

In a previous study, we reported that AC power supply treatment did not affect yeast cell viability and OD_600_ measurement, which proved the safety of AC-driven plasma jet for yeast cells [15]. Fig 8A and 8B illustrate the impact of a pulsed DC-ignited jet on yeast cells in a culture medium. The results show that cell viability and growth are not significantly altered. The error bars in Fig 8 represent the standard deviation of the mean calculated from three independent measurements for Condition A, and four independent measurements for Condition B.

**Fig 9:**
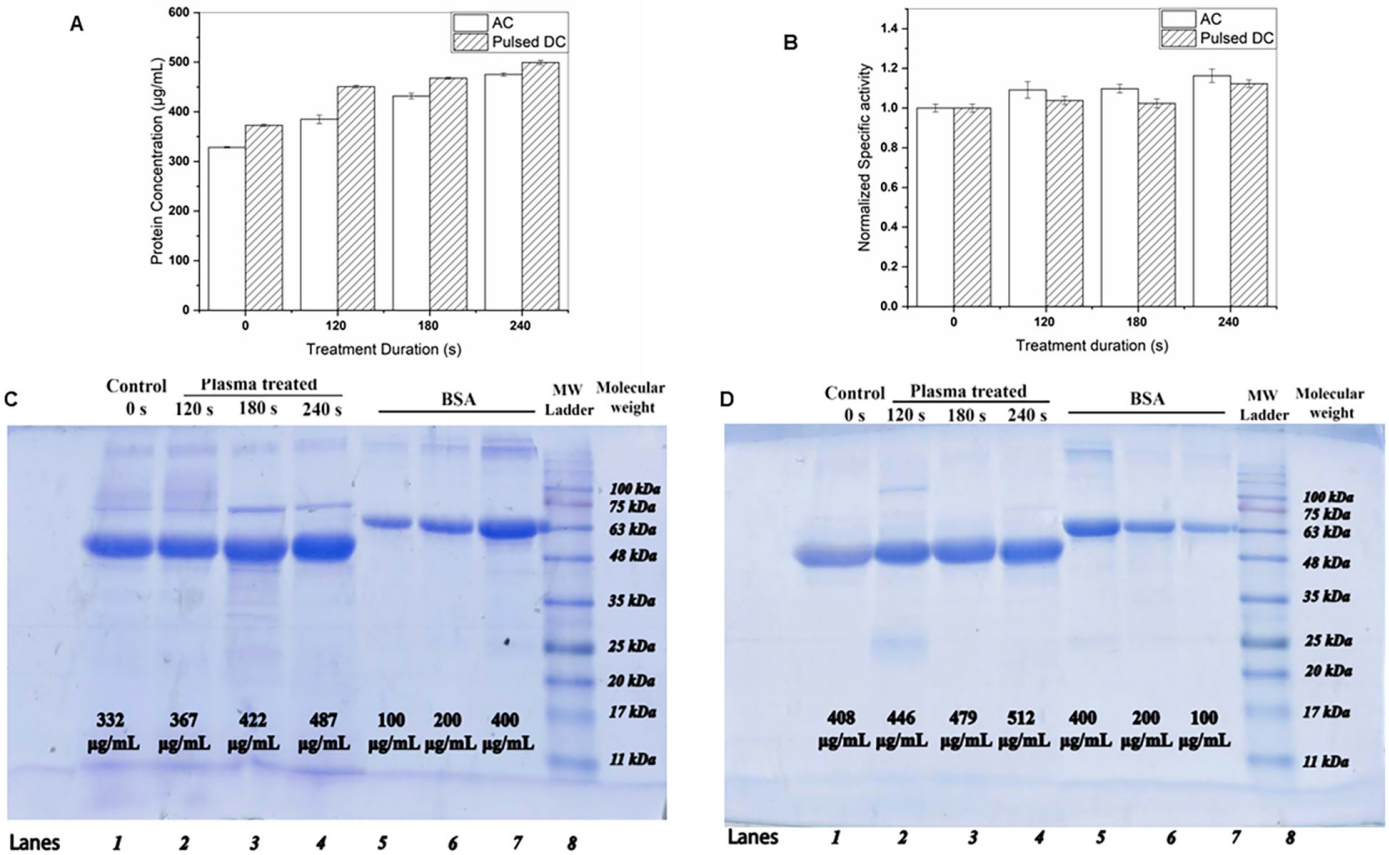
Total protein concentration of culture medium and activity after treatment by AC/Pulsed DC-driven plasma jet evidenced an increasing trend proportional to the treatment time. (A) Total protein concentration by Bradford assay (B) Normalized enzyme activity colorimetric measurement. The error bars in the plots represent standard deviations. Total protein concentration by estimation through ImageJ densitometry of SDS-PAGE gel image in (C) AC mode, and (D) Pulsed DC mode (Lines 1–4 collected supernatants, lines 5–7 BSA standards, line 8 molecular ladder).

A Bradford assay was performed on the culture medium to assess the effect of plasma duration treatment on protein production. Protein concentrations were quantified through absorbance measurements at 595 nm and comparison to a standard curve of bovine serum albumin. As depicted in Fig 9A, both AC pulsed DC plasma configurations resulted in increased protein production relative to untreated controls, with the pulsed DC plasma yielding higher protein levels. The error bars represent the standard deviation of the mean calculated from four independent repeated measurements.

The results showed that both sets of data demonstrate an increase in protein production that is dependent on the dose of plasma treatment, which is equivalent to the duration of the treatment. The increase was observed to be up to 45% and 33% in the plasma jet ignited by AC and pulsed DC, respectively. This implies that both AC and pulsed DC power supplies can stimulate yeast cells to produce more protein, however; the AC arrangement has shown a slightly more productive result.

The results of the normalized enzyme activity (U/ml) measured using a DNS-based colorimetric protocol after the enzyme’s action on CMC polymer are presented in Fig 9B as column plots. The error bars represent standard deviations from the means of four independent measurements. The findings indicate that a 1.25-fold multiplication of enzyme activity for maximum treatment is detected for the AC operation. The increase percentage for pulsed DC was a bit smaller, 1.19. Overall, plasma induction has increased enzymatic activity.

Fig 9C and 9D show SDS-PAGE images after Coomassie blue staining, which illustrate sharp bands of EgII (lanes 1 to 4) at 48 kDa after two different plasma induction, along with BSA bands (lanes 5 to 7) at the 66.5 kDa position. The ImageJ analysis of gel images provides an estimation of expressed concentrations via densitometry. The results exhibit the same trend as the Bradford measurement. The protein production has grown by plasma treatment with an increase in treatment duration. The DBD jet in both modes was able to stimulate the yeast cell for more recombinant protein production, possibly through a chain of reactions in metabolism. A growth percentage of measured protein concentrations can be inferred from the estimated protein densities. The percentages were 47% for AC-driven jet, and 39% for DC-operated plasma jet.

## 4. Discussion

Plasma medicine research has shown that plasma can generate RONS that can trigger specific pathways in cells [15, 38, 39]. Specifically, the RONS induce the activation of stress response pathways in cells, which, depending on the CAP intensity and cell type, can either be advantageous for cell survival or improvement or lead to cell death [40].

DBD configurations of plasma at the kHz range result in the accumulation of charge on the dielectric surface. This accumulation of charge leads to the electric field diminishing, which ensures the low temperature of the plasma plume that can be applied to cells [12]. Sinusoidal AC voltage power supplies are widely used in the industry due to their low current and power dissipation. On the other hand, the DC bias of power supplies is pulsed to reduce direct and high currents, thereby preventing harm to the target. However, the higher current of DC pulsed bias compared to AC bias may be a potential drawback in cell-based studies [41].

The purpose of the present analysis was to gain a better understanding of plasma dynamics and compare the performance of two power supplies in recombinant protein production. The investigation was conducted on both modes of a single plasma jet to ensure the safety of CAPJ in a live model organism (a recombinant yeast *Pichia pastoris*). *Pichia pastoris* cells were induced for protein production and then immediately exposed to the plasma treatment, driven by either an AC field or pulsed DC bias, for 120, 180, and 240 seconds. The presented results suggest that the atmospheric DBD plasma jet has a constructive role in gene expression, and that plasma exposure does not negatively affect cell viability or growth. These outcomes indicate that CAP is a robust and scalable tool in the recombinant protein industry. Therefore, it’s crucial to measure and control the level of reactive species in plasma for proper dosage and positive impressions. Low doses of reactive species generated by CAP exposure can affect the gene expression profile of treated cells, which may include an increase in the expression of recombinant protein production [42].

In a previous study, the authors investigated oxidative stress and found that short plasma induction has regulatory effects, significantly upregulating oxidative stress genes, including catalase A (CTA1), superoxide dismutase (SOD1), and yeast transcription factor (YAP1). These upregulations are believed to be beneficial in providing elevated energy deposits for recombinant protein production [15, 43]. The upregulation persisted for up to 24 hours for some genes, which is a synergistic achievement of plasma-generated stable species like hydrogen peroxide and intracellular production of RONS. In other words, exposure to RONS can initiate counter-regulation processes in cells to maintain redox balance [44, 45].

The findings were consistent with other studies that demonstrate a biphasic cell response based on the Hormesis model [26, 46]. The model suggests that low doses of stressors can induce peculiar phenomena with regulating and signaling results, but high doses will overburden the scavenging agents and may initiate cell death [26, 38].

The characteristics of cold plasma, such as treatment duration, input/output voltage, flow rate, and composition of the feed gas, can be varied to obtain different major species. The collective features are referred to as the "plasma dosage," which is a key factor in the Hormesis model [47, 48]. Electrons play a significant role in plasma reactions, and therefore, it is essential to estimate their density and temperature. Here, a 2D FEM simulation of fluid dynamics and gas discharge was performed to gain insight into the events related to electrons. Simulations provided interesting patterns of n_e_, temperature, and plasma ionization waves for both modes. Furthermore, a roughly approximated T_e_ and n_e_ was determined using the line ratio method, applied to selected lines of the recorded OES from plasma ignited by a sinusoidal AC power supply.

The n_e_ is an important contributor to ionization, plasma chemistry, and reactive species production [49, 50]. According to simulation outputs, aggregations of electrons are formed around the inner electrode by voltage application to generate the avalanches and subsequent discharge. The COMSOL computations provided n_e_ to be 10^20^−10^23^ m^-3^ for pulsed operation and 10^20^−10^21^ m^-3^ for AC mode. The elevated density in a DC-driven jet, up to 10^23^ m^-3^, explicitly transient, makes the average quantity superior to its counterpart in AC mode. The difference in electron densities between the two jets is caused by the higher voltage applied to the jet during pulsed operation. The applied voltage in sinusoidal mode is 4 kV, whereas it is 10 kV in pulsed mode. It is important to note that according to 2% duty cycle characteristics of pulsed DC power supply, the plasma is only turned on for two microseconds during the 100-microsecond time frame, which may explain the better efficiency of AC mode despite the higher electron densities of DC power supply that guarantee active plasma chemistry. Besides, calculated n_e_ from the line ratio method for a pair of helium peaks is a good agreement with the COMSOL simulation. However, caution should be taken when applying the method, as the chemistry of mixtures containing nitrogen is complicated, necessitating consideration of vibrational exchange processes between vibrational levels of nitrogen and atoms and molecules [51, 52].

It’s well accepted that the electric discharge in the plasma jet is composed of a train of high-speed traveling bullets, as observed for the first time by Teschke et el. in 2005 and later by other researchers [53, 54]. The plasma plume is visible to the naked eye as a continuous luminous flow [55], however, bullets are not observable without the aid of high-speed cameras [54, 56, 57]. The ionization wave front in the form of bullets was eye-catching in both cases of simulation, representing a maximum abundance at the bullet center. Evidence of the ionization wave’s propagation was provided in Figs 5 and 6 by time snapshots of n_e_, obtained from simulations. Even in transient pulsed mode, the propagation of bullets won’t stop. The space charge resulting from charge deposition on the dielectric surface generates an opposing electric field with regard to the applied electric field, as mentioned above. If the electric field becomes high enough, it will have strength for a new breakdown formation, and a second discharge can occur after the initial pulse-induced discharge and sustain the plasma plume even after the pulse ends [58]. Note that the n_e_ on the dielectric surface is low compared to the discernible, highly ionizing region.

The T_e_ is another important parameter of plasma that represents the ability for ionization, excitation, and dissociation processes. Although there are many methods to determine T_e_ in plasma diagnostics [59], the simple line ratio method was applied in the current study to select lines of nitrogen in the recorded spectrum of AC mode. Inserting calculated density from Eq 1 into Eq 2 resulted in a 1.4 eV temperature, coinciding with the COMSOL simulations. Fig 4 exhibited the modeled T_e_ with almost uniform energy distribution in both jet ignitions, except for the rod electrode neighborhood.

The present study confirms that both investigated power supplies could influence the recombinant yeast in the oxidative eustress range, instigating a prosperous protein yield. In summary, the AC power supply provides a lower number of electrons at higher temperatures, while the more populated pulsed DC induction has been hindered from achieving its expected efficacy due to the long off-state duration in the pulse. Nevertheless, n_e_ and temperature are high during the applied voltage pulse in pulsed mode, but the average quantities are lower.

Specifically, the AC-driven plasma jet has been successful in enzyme production and activity improvement, while the pulsed operation of the DC power supply has relieved the target from high current, making it a potential choice for cell modifications by CAP.

Time-resolved imaging by a high-speed intensified charge-coupled device (ICCD) camera can provide practical insights about plasma dynamics and validate plasma simulations visually. A single jet is not efficient considering the mass production complications in biotechnology industries. However, predictions can be made that an array of optimized plasma jets applied to a fermentor would provide a worthy productive outcome.

## Supporting information

S1 File The files include excel files of Bradford, MTT, enzyme activity assays, optical density measurements, optical emission spectrum, and a PDF file composed of original images of gels. (RAR)

S1 Raw images (PDF)
