# Supplementary figures and images for: Simulation and experimental study of a cold atmospheric pressure plasma and comparison of efficiency in boosting recombinant Endoglucanase II production in Pichia pastoris
Source: PLoS One. 2024 May 21;19(5):e0303795. doi: 10.1371/journal.pone.0303795 (PMC11108213; doi:10.1371/journal.pone.0303795)

**Control**

0 s

**Plasma treated**

120 s

180 s

240 s

**BSA**

**MW**

Ladder

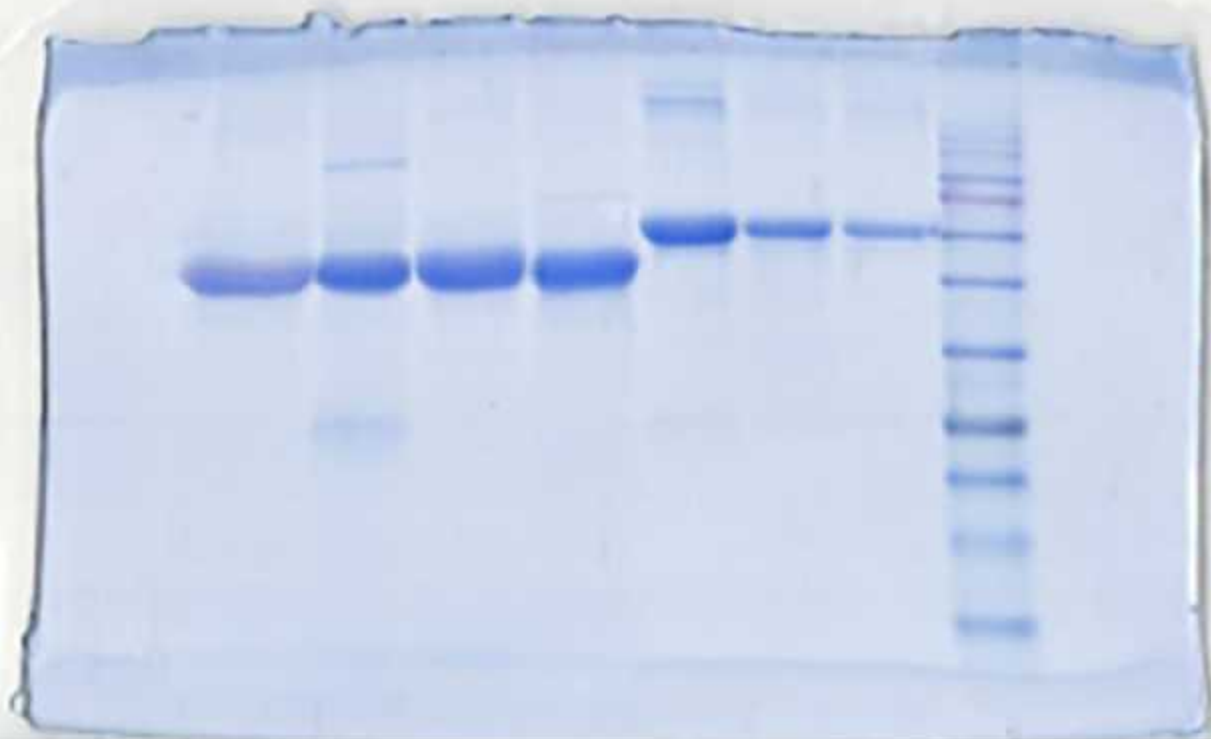

| Control | Plasma treated |       |       | BSA |  | MW     |
|---------|----------------|-------|-------|-----|--|--------|
| 0 s     | 120 s          | 180 s | 240 s |     |  | Ladder |

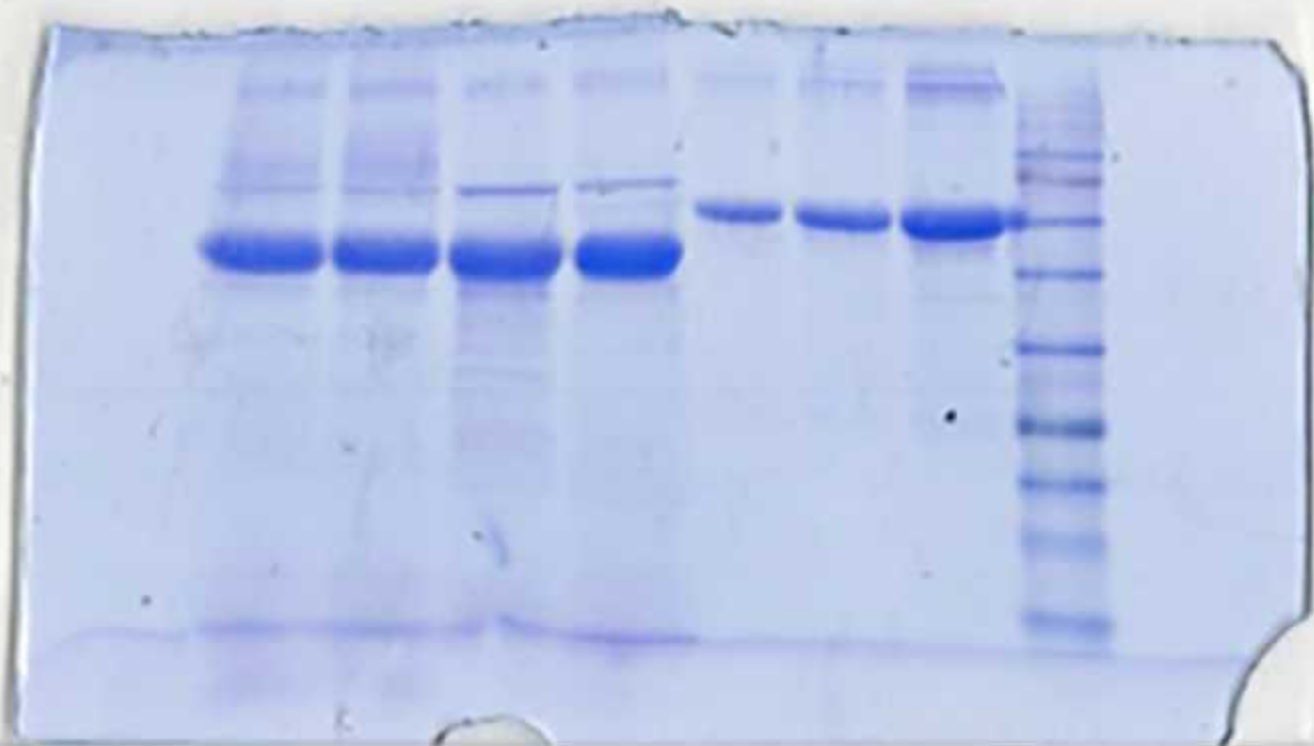

Supplement: S1 Raw images — (PDF) [file pone.0303795.s002.pdf]
